# Combined effects of ZnO particle deposition and heat treatment on dimensional stability and mechanical properties of poplar wood

**DOI:** 10.1038/s41598-017-10606-5

**Published:** 2017-08-30

**Authors:** Wei Cui, Nannan Zhang, Min Xu, Liping Cai

**Affiliations:** 1 Key Laboratory of Bio-based Material Science & Technology (Northeast Forestry University), Ministry of Education, Harbin, 150040 P.R. China; 2 Mechanical and Energy Engineering Department, University of North Texas, Denton, Texas 76201 USA

## Abstract

This study proposed a one-step wood modification method by combining the deposition of ZnO particles on wood surface and heat treatment. The effects of ZnO particles and heat treatment on mechanical properties and dimensional stability of poplar wood were examined. Samples were sorted into 4 groups, i.e., control, heat-treated, impregnation/heat-treated, and hydrothermal-treated samples. The mechanical properties and dimensional stability of impregnation/heat-treated and hydrothermal-treated wood samples were measured in comparison with those of the control and heat-treated wood samples. Compared with the control ones, the reduction of the flexural strength of the heat-treated, impregnation/heat-treated and hydrothermal-treated samples were about 11.57%, 8.53% and 15.90%, respectively. The modulus of elasticity of the heat-treated and hydrothermal-treated samples decreased by 13.78% and 23.78%, respectively, while the impregnation/heat-treated samples increased by about 8.57% due to the ZnO particles growth. The dimensional stabilities of the heat-treated, impregnated/heat-treated and hydrothermal-treated samples were improved in comparison with that of the control sample. All samples were characterized by the scanning electron microscopy (SEM), Fourier transform infrared spectroscopy (FTIR) and X - ray diffraction (XRD).

## Introduction

Heat treatment is considered as an eco-friendly modification process, which improves some wood properties, referring to the enhanced hygroscopic properties and dimensional stability^1^, decreased wettability^2^, and increased durability^3^. However, the drawbacks of heat treatment are that the modulus of rupture (MOR) and modulus of elasticity (MOE) are reduced^4, 5^. During the heat treatment, the chemical composition and structure of the wood^6^ were changed, resulting in a mass loss^7^. With the increases in temperature and time of the heat treatment, the compression strength^8^ and the density of the wood decreased^9^. Therefore, it is necessary to compensate for the decrease in wood mechanical properties during heat treatment.

A series of physical and chemical methods have been proposed to alleviate the negative effects of heat treatment, such as integrating heat treatment with densification^10^, borate salt^11^ and silver nanopaticles treatments^12^, but these methods need complex process and difficile-manipulation. Another approach was to deposit the nanomaterials on wood surfaces using the hydrothermal method^13^, for instance, sedimentation of ZnO nanorods, TiO_2_/Cu_2_O composite coating on the wooden substrate^14, 15^. The hydrothermal methods can be described as the use of high temperature, high pressure aqueous solution to make the normally soluble or insoluble materials dissolved and re-crystal^16^. However, the hydrothermal reaction is carried out at a high temperature and high pressure consuming great amounts of energy, and an expensive sealed autoclave is a prerequisite for the hydrothermal reaction^17^. Meantime, the preparative methods for forming particles on wood surfaces are generally complex and include at least two or three steps^18^, which limits its application in the large sized wood modification.

The objective of this study was to develop a one-step wood modification method by combining the deposition of ZnO particles on wood surfaces and heat treatment to alleviate the mechanical property loss caused by heat treatment. The microcosmic characterization and formation mechanism of ZnO particles of the impregnation/heat treatment were discussed in comparison with the hydrothermal treatment. The examination results of mechanical properties and dimensional stability showed that the impregnation/heat treatment generating zinc oxide on the wood surface compensated the loss of mechanical properties that caused by the heat treatment, and the dimensional stability was improved in comparison with the heat-treated poplar.

## Results and Discussion

**Figure 1:**
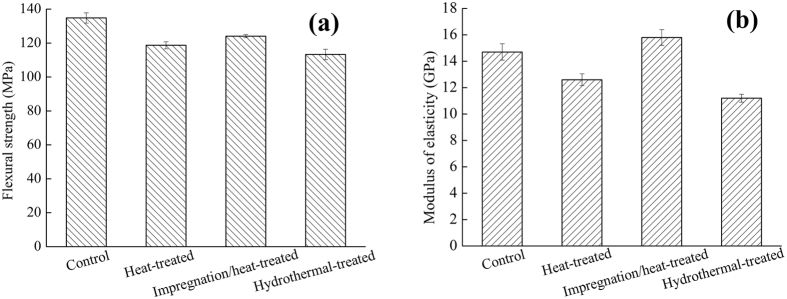
Flexural strength (**a**) and Modulus of elasticity (**b**) of wood.

Figure 1 shows the flexural strength and modulus of elasticity of the control, heat-treated, impregnation/heat-treated and hydrothermal-treated samples. Compared with the control samples, the reduction of the flexural strength of the heat-treated, impregnation/heat-treated and hydrothermal-treated were 11.94%, 7.94%, and 19.19%, respectively, indicating that the impregnation/heat-treated method reduced the loss of flexural strength. Compared with the control samples, MOE of the heat-treated and hydrothermal-treated samples decreased by 14.29% and 23.81%, respectively, while the MOE of the impregnated/heat-treated samples increased by 7.48%.

**Figure 2:**
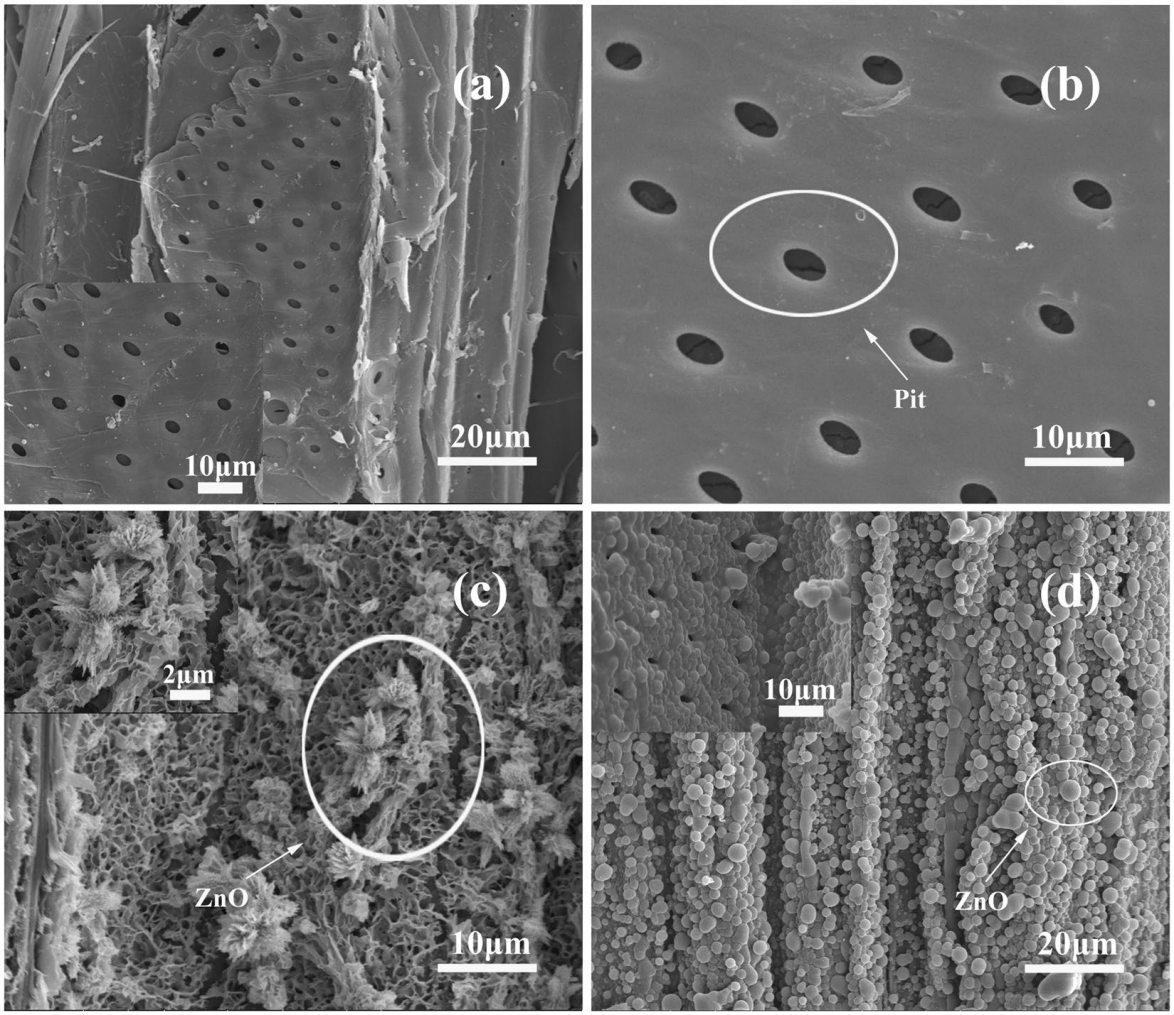
SEM images of (**a**) Control, (**b**) Heat-treated (**c**) Impregnation/heat-treated, (**d**) Hydrothermal-treated.

The fact of the reduction of the flexural strength due to the heat-treatment was consistent to that of previous studies^19–21^, which could be reasoned by the degradation of hemicelluloses in the high temperature process^22^. The impregnation/heat treatment generating zinc oxide on the wood surfaces could compensate the loss of mechanical properties that caused by the heat treatment. It was probably because of the zinc hydroxides deposited in wood catheters and cell lumens during the process of impregnation/heat treatment as shown in Fig. 2(c). Since the Zinc hydroxide could be easily dehydrated to produce Zinc oxide particles at 170 °C, a large amount of Zinc oxide particles grew in wood catheters, which increased the surface density of the wood. At the same time, the rigidity of the wood increased in high temperatures, and the combined effects made a compensation for the loss of mechanical properties during the heat treatment. As for the hydrothermal method, it was possible due to the high relative humidity^23^ of the treatment environment to accelerate the degradation of hemicelluloses, resulting in more significant reduction of flexural strength and MOE than that of the heat-treated wood.

Figure 2(a) shows the surface morphology of the control samples, illustrating that the wood surface was smooth with some clearly visible pits. Figure 2(b) presents the surface morphology of the heat-treated samples, which also have some visible pits, illustrating that no significant change occurred compared with that of the control ones. Figure 2(c) shows the surface morphology of ZnO particles after the impregnated/heat treatment. In the upper left corner in Fig. 2(c), it can be found that the flower-like ZnO crystal was composed of small-sized needle and sheet-like ZnO. This may be due to heat treatment conditions. The heat and mass transfer was more intense than that samples treated by the hydrothermal method, resulting in the faster Zn(OH)_2_ dehydration reaction, sharper formation of ZnO and more uneven appearance. The formation of ZnO increased the wood surface roughness, resulting in the improvement of dimensional stability of wood. Figure 2(d) shows that the surface morphology of the wood was covered by ZnO particles after the hydrothermal treatment. The ZnO-treated surface was uniform and the particles were compact and spherical, suggesting that ZnO particles can be formed on the wood surface by the hydrothermal method under certain reaction conditions. This was mainly due to the rich hydroxyl on wood surface and ZnO on the hydrophilic wood substrate. The ZnO particles can effectively nucleation in cellulose fiber and the nuclear began to grow.Therefore, when the wood was immersed in the reaction solution, the Zn^2+^ in the precursor solution was connected to the surface of the wood by the hydrogen bonding energy under the action of hydrothermal energy. As the reaction time prolonged, Zn^2+^ was aggregated into ZnO particles. As the continuous action of hydrothermal energy continued to grow, the self - assembly of surface activity continued on the wood surface.

**Figure 3:**
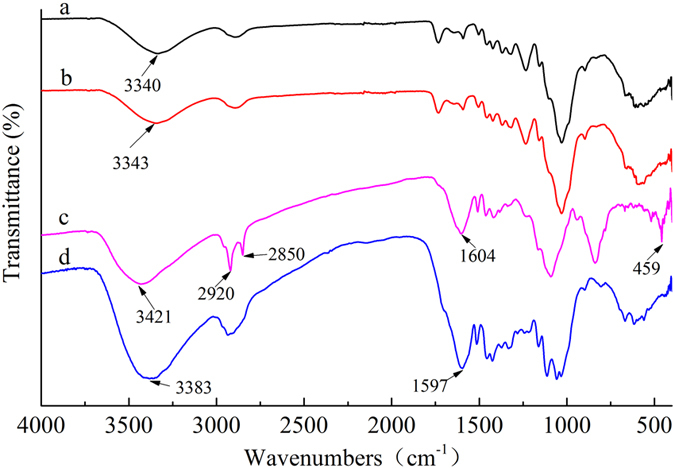
FTIR spectra of wood: (**a**) Control, (**b**) Heat-treated, (**c**) Impregnation/heat-treated, (**d**) Hydrothermal-treated.

Figure 3 shows the FTIR spectra of the control, heat-treated, impregnation/heat-treated and hydrothermal-treated samples. The peaks near 3400 cm^−1^ was assigned to stretching vibrations of hydroxyl groups on the wood surface, in which, the band at 3340 cm^−1^ (a curve) and 3343 cm^−1^ (b curve) corresponding to the stretching vibrations of hydroxyl groups in the wood shifted to larger wavenumbers of 3421 cm^−1^ (c curve) and 3383 cm^−1^ (d curve), indicating a strong interaction between the hydroxyl groups of the wood surface and ZnO particles through hydrogen bonds^24^. For impregnation/heat-treated samples, the two strong absorption peaks at 2920 cm^−1^ and 2850 cm^−1^were ascribed to the asymmetrical stretching vibrations of −CH_3_ and −CH_2_^25^. New bands at 1604 cm^−1^ (c curve) and 1597 cm^−1^ (d curve) appeared in the spectrum of the ZnO particles deposited wood, corresponding to the asymmetric and symmetric stretching of zinc carboxylate at the surface of the ZnO particles^14^. In impregnation/heat-treated samples, the characteristic absorption peak of ZnO appeared around 459 cm^−1^, indicating that the wood surface had the ZnO formation after the impregnation/heat treatment^26^.

**Figure 4:**
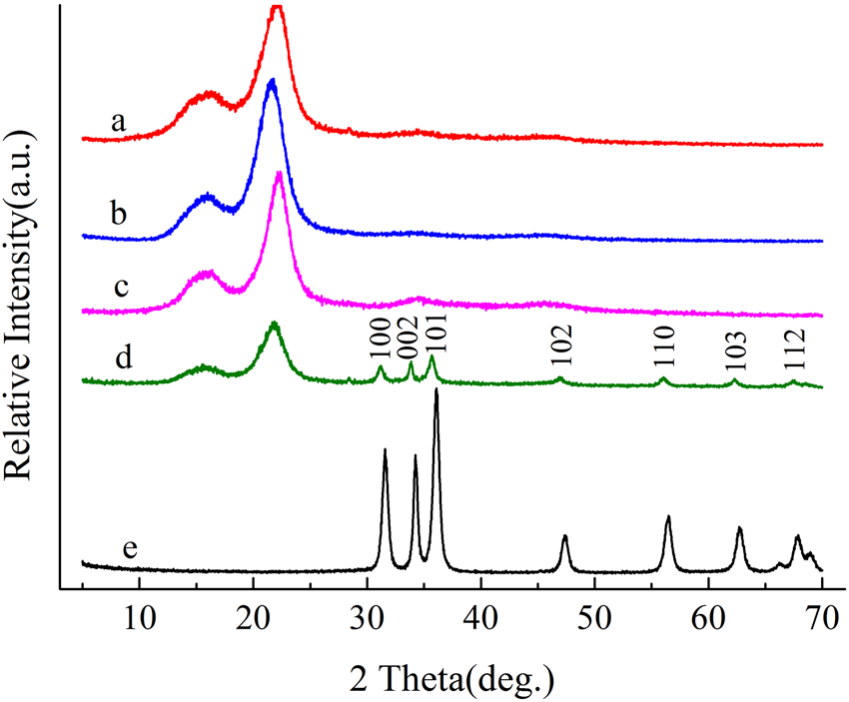
XRD patterns of (**a**) Control, (**b**) Heat-treated, (**c**) Hydrothermal-treated, (**d**) Impregnation/heat-treated, (**e**) ZnO.

Figure 4 shows the XRD patterns of the control, heat-treated, hydrothermal-treated, impregnation/heat-treated and ZnO samples. In Fig. 4, the characteristic diffraction peak of cellulose (2θ = 15, and 22°) appeared in the control, heat-treated, impregnation/heat-treated and hydrothermal-treated samples, indicating that the crystal form of cellulose was not changed^27^. The characteristic diffraction peaks of cellulose and ZnO diffraction peaks were observed at the same time in the impregnation/heat-treated samples. It was also found that the characteristic diffraction peak intensity of the cellulose modified by the impregnated-heat treatment was weaker than that of the control and heat-treated samples. The location of the diffraction peaks of ZnO in the XRD patterns of the impregnation/heat-treated sample was consistent with the diffraction peak position of pure ZnO. It can be seen that the ZnO-formed on wood surface was hexagonal wurtzite structure (JCPDS card No. 36-1451)^28^. The relatively strong and sharp XRD diffraction peaks showed that the ZnO crystals formed in the wood were well crystallized. Therefore, it can be deduced that the particles formed on the wood surface by the impregnation/heat treatment were pure ZnO crystals.

**Figure 5:**
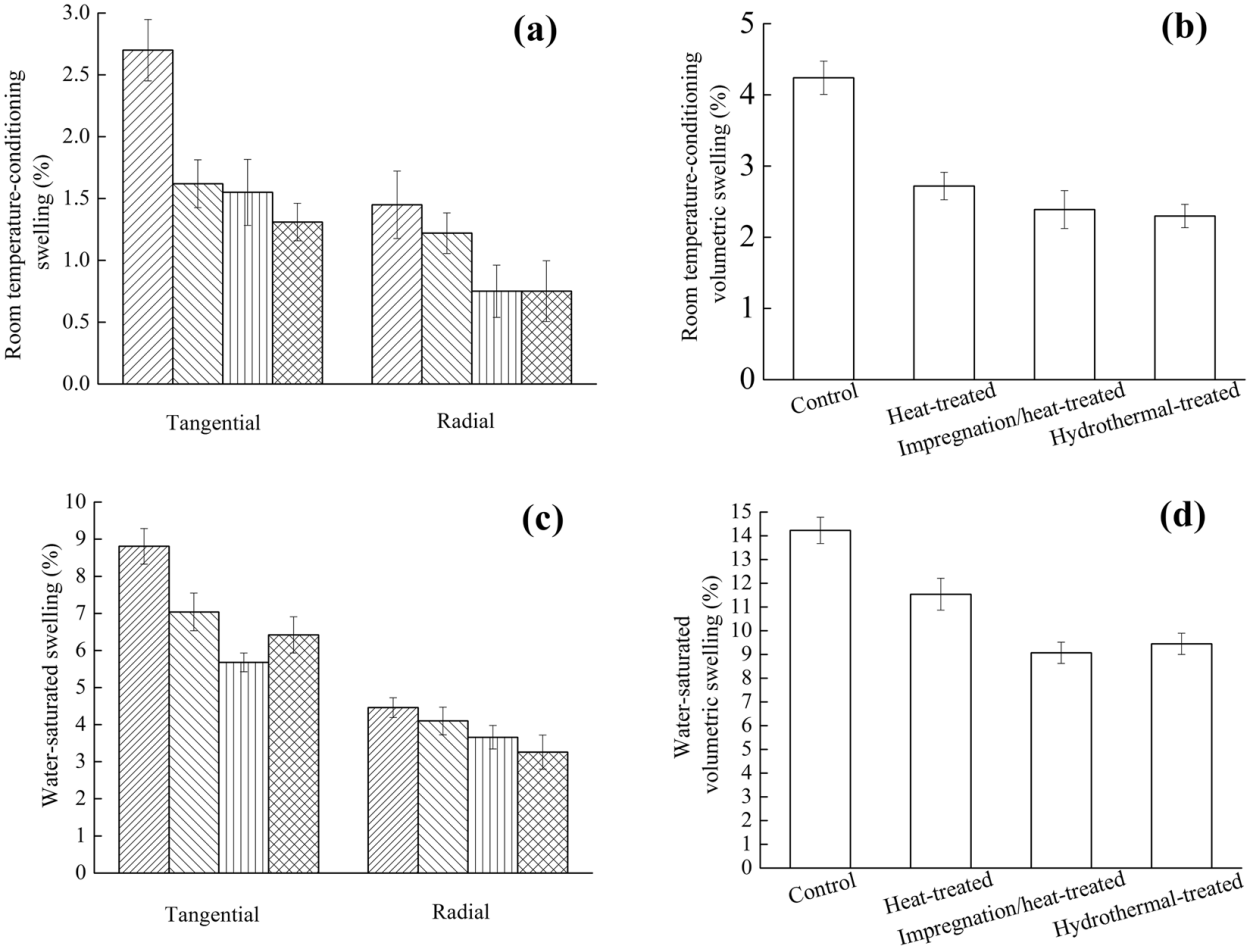
Room temperature-conditioning swelling of wood: (**a**) Tangential and Radial, (**b**) Volumetric. Water-saturated swelling of wood: (**c**) Tangential and Radial, (**d**) Volumetric.

After measuring the dimensions of oven-dried samples, the samples were placed in a room with a temperature of 20 ± 2 °C and a relative humidity of 65 ± 3%. The samples swelled as moisture content increased until the dimensional swellings stopped, and the dimensions were measured again to obtain the room temperature-conditioning swelling. The swellings of the wood samples after different treatments are shown in Fig. 5. It can be seen in Fig. 5(a) that the tangential and radial swellings of the heat-treated, the impregnated/heat-treated and the hydrothermal-treated samples were reduced by different percentages compared with that of the control samples. Compared with the control samples, the tangential swellings of the heat-treated, impregnation/heat-treated and hydrothermal-treated samples were reduced by 40.00%, 42.59%, and 51.48%, respectively. The radial swellings of the heat-treated, impregnation/heat-treated and hydrothermal-treated samples were reduced by 15.81%, 48.28%, and 48.28%, respectively. As shown in Fig. 5(b), the reductions in volumetric swelling were achieved by heat-treated, impregnation/heat-treated and hydrothermal-treated. Compared with the control samples, the volumetric swellings of the heat-treated, impregnation/heat-treated and hydrothermal-treated samples were decreased by 35.85%, 43.63%, and 45.75%, respectively. According to these results, it was confirmed that the depositing of ZnO particles can reduce the moisture absorption of wood.

After measuring the dimensions of oven-dried samples, the samples were immersed in distilled water at a temperature of 20 ± 2 °C for 20 days until the stable dimensions were obtained. The dimensions were measured to obtain the water-statured swelling. Figure 5(c) shows that, compared with the control samples, the tangential swellings of the heat-treated, impregnation/heat-treated and hydrothermal-treated samples were reduced by 20.09%, 35.52%, and 27.13%, respectively. The radial swellings of the heat-treated, impregnation/heat-treated and hydrothermal-treated samples were reduced by 8.07%, 17.93%, and 26.91%, respectively. As shown in Fig. 5(d), compared with the control samples, the volumetric swellings of the heat-treated, impregnated/heat-treated and hydrothermal-treated samples were reduced by 18.9%, 36.26%, and 33.59%, respectively.

**Figure 6:**
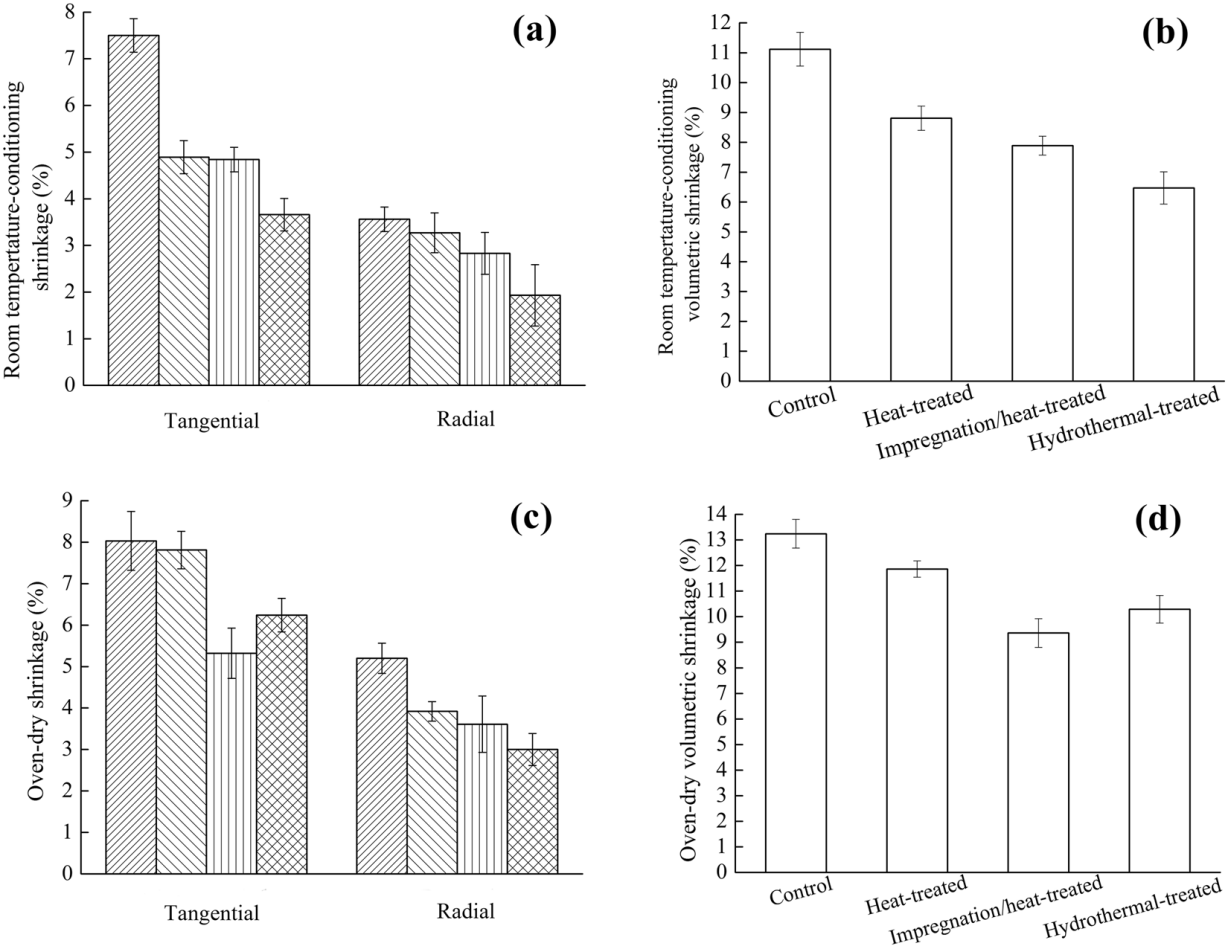
Room temperature-conditioning shrinkage of wood: (**a**) Tangential and Radial, (**b**) Volumetric. Oven-dry shrinkage of wood: (**c**) Tangential and Radial, (**d**) Volumetric.

After water-saturated swellings were measured, the samples were dried under the conditions of the temperature of 20 ± 2 °C and relative humidity of 65 ± 3%, and the shrinkages were measured as the dimensions were stable. The room temperature-conditioning shrinkages of wood samples by different treatments are shown in Fig. 6(a,b). As shown in Fig. 6(a), compared with the control samples, the tangential shrinkages of heat-treated, impregnated/heat-treated and hydrothermal-treated samples decreased by 34.80%, 35.47%, and 51.20%, respectively. The radial shrinkages of the heat-treated, impregnation/heat-treated and hydrothermal-treated samples decreased by 8.15%, 20.51%, and 45.79%, respectively. Figure 6(b) shows that the volumetric shrinkages of the heat-treated, impregnation/heat-treated and hydrothermal-treated samples were lower than that of the control samples. Compared with the control ones, the volumetric swellings of the heat-treated, impregnated/heat-treated and hydrothermal-treated samples were reduced by 20.77%, 29.05%, and 40.82%, respectively.

After the water-saturated dimensions were measured, the samples were dried in an oven with a temperature of 103 °C until the constant weights were obtained. The oven-dried shrinkages were calculated and are illustrated in Fig. 6(c,d). As shown in Fig. 6(c), compared with the control samples, the tangential shrinkages of heat-treated, impregnated/heat-treated and hydrothermal-treated samples decreased by 2.74%, 33.75%, and 22.29%, respectively. The radial shrinkages of the heat-treated, impregnation/heat-treated and hydrothermal-treated samples decreased by 24.62%, 30.58%, and 42.31%, respectively. Figure 6(d) shows that, compared with the control samples, the volumetric shrinkages of the heat-treated, impregnated/heat-treated and hydrothermal-treated samples decreased by 10.42%, 29.31%, and 22.28%, respectively.

By taking the swelling and shrinkage into account, the results showed that the dimensional stability of the heat-treated, impregnation/heat-treated and hydrothermal-treated samples were improved in comparison with that of the control ones. Compared with the heat-treated samples, the dimensional stability of the two modified samples was also improved. There were two main reasons for the improvement. Firstly, hemicellulose degraded in high temperature conditions, resulting in the decrease of free hydroxyl groups, improving the dimensional stability of wood^29^. Secondly, as shown in Fig. 2(c,d), the ZnO particles on the wood surface deposited by the impregnation/heat-treatment and hydrothermal-treatment, which formulated an efficient coating for suppressing water absorptions^30^, improving the dimensional stability of the wood.

## Conclusion

ZnO particles were successfully deposited on wood surfaces by one-step impregnation/heat treatment method.

1. The impregnation/heat treatment generating zinc oxide on the wood surface compensated the loss of mechanical properties that caused by heat treatment. Compared with the control ones, MOE of the heat-treated and hydrothermal-treated samples decreased by 14.29% and 23.81%, respectively, while the impregnation/heat-treated samples increased by about 7.48% due to the ZnO growth.
2. The dimensional stabilities of the heat-treated, impregnated/heat-treated and hydrothermal-treated wood were improved in comparison with that of the control samples. The dimensional stabilities of the impregnated/heat-treated and hydrothermal-treated samples were also improved compared with that of the heat-treated wood, indicating that the deposition of ZnO particles on wood surface enhanced wood resistance to moisture.
3. After the impregnation/heat treatment, the wood surface of the zinc oxide showed flower-like crystals. The examination of SEM, FTIR and XRD confirmed that he zinc oxide particles could be grown on wood surface by the one-step impregnation/heat treatment method, which caused the structural changes of the functional groups.

The one-step impregnation/heat treatment method simplified the processing steps of the deposition of ZnO particles on wood surface and improved the energy efficiency.

## Materials and Methods

### Materials

The Poplar (*PopulusAdenopodaMaxim*) wood was obtained from Mudanjiang, Heilongjiang Province, China. The Zinc acetate, with a purity ≥ 99%, was supplied by Tianjin Zhiyuan Chemical Reagent Co., Ltd. The Sodium hydroxide particles (analytical grade) were supplied by Tianjin Tianli Chemical Reagent Co, Ltd. The Ammonia, with a concentration of 25%, was supplied by Tianjin Quartz Clock Factory Bazhou City Chemical Factory.

### Methodology

**Figure 7:**
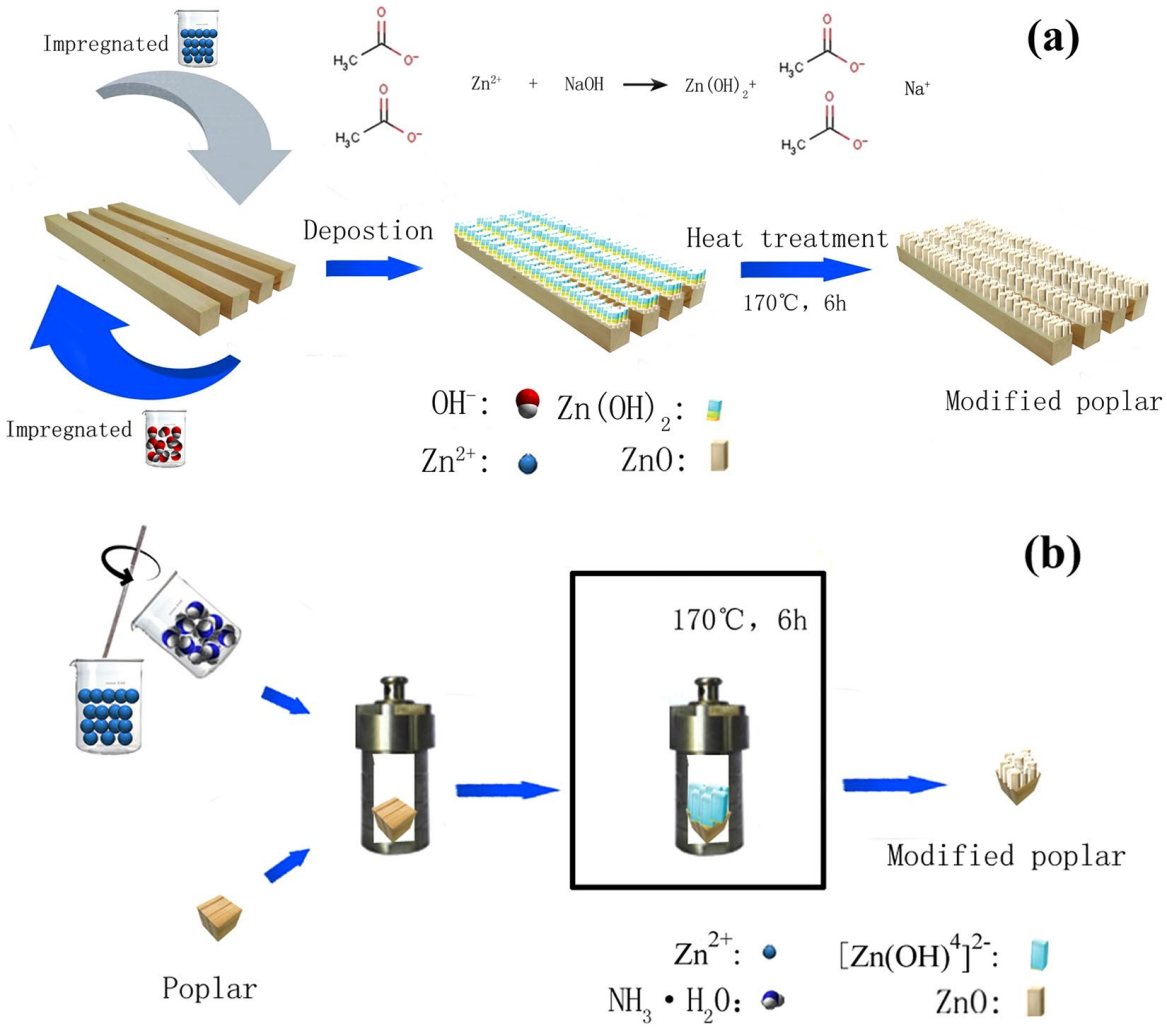
Schematic illustration of: (**a**) Impregnation/heat treatment and (**b**) Hydrothermal treatment.

The process of depositing zinc oxide on wood surface by impregnation/heat treatment is presented in Fig. 7(a). A certain amount of 1 mol / L Zn (Ac)_2_ and NaOH solution were prepared firstly. The wood samples were immersed in the solution of Zn (Ac)_2_ and remained at a vacuum of 0.01 MPa absolute pressure, (75 Torr) for 8 hours and then dried in a drying oven at 60 °C. The wood samples were taken out and immersed in the NaOH solution for 8 hours in the vacuum pressure, and then dried in a drying oven at 60 °C. After the impregnation processes of Zn (Ac)_2_ and NaOH, the unreacted chemical substances were removed from the wood sample surfaces using the deionized water. The ZnO-deposited wood samples were heat-treated at 170 °C for 6 h. The process of generating zinc oxide on poplar surfaces by hydrothermal treatment is presented in Fig. 7(b). The major factors influencing the ZnO formation morphology on the surface of poplar were the reaction temperature, reaction time and reaction solution Zn^2+^/OH^−^ ratio. Based on the single factor test, the optimum conditions ZnO morphology of poplar surface were as follows: the reaction temperature of 170 °C, reaction time of 6 h, and solution Zn^2+^/OH^−^ ratio of 1: 1. Zn (Ac)_2_ • 2 H_2_O (analytically grade) was dissolved in the deionized water. After stirring at room temperature for 30 min, a certain amount of NH_3_ • H_2_O was added and stirred for 10 min. The resulted solution and the wood samples were transferred to a reactor equipped with a polytetrafluoroethylene liner. The reaction was carried out at 170 °C for 6 h and then the samples were removed out from the reactor and cooled to room temperature. The wood samples were washed three times with deionized water to remove unreacted chemicals and residues on the wood surfaces and then dried at 60 °C for 24 h. The heat treatment were performed at 170 °C for 6 h using a temperature-controlled laboratory oven.

### Mechanical property tests

From each treatment group, six samples sized 80 mm × 13 mm × 4 mm were prepared for examining the flexural strength and MOE by means of a universal testing machine (Ruigeer, RGT-20A) in accordance with the procedure described in the ASTM D 7031 standard. Three-point bending set-up was used with a span of 64 mm and a crosshead speed of 2.5 mmmin^−1^.

### Dimensional stability tests

**Figure 8:**
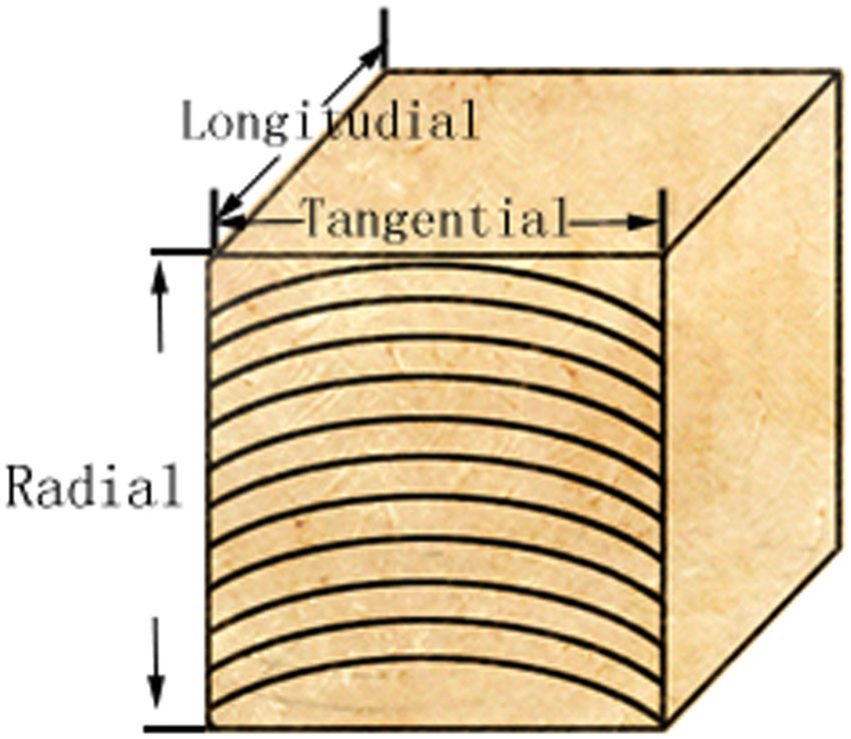
Schematic illustration of dimensional stability measurement.

From each treatment group, forty samples sized 20 mm × 20 mm × 20 mm were prepared for four different tests as follows. a) Room temperature-conditioning swelling: the oven-dried samples were placed under the condition of a temperature of 20 ± 2 °C and relative humidity of 65 ± 3% until the dimensions were stable. b) Water-saturation swelling: the oven-dried samples were immersed in distilled water at a temperature of 20 ± 2 °C for 20 days until the stable dimensions were obtained. c) Room temperature-conditioning shrinkage: the water-saturated samples were placed at the condition of the temperature of 20 ± 2 °C and relative humidity of 65 ± 3% until the dimensions were stable. d) Oven-dry shrinkage: the water-saturated samples were placed in an oven with a temperature of 103 °C and dried until the constant weight was obtained (bone dried). During the four tests, the dimensions were measured in three directions (longitude, radius, and tangential as shown in Fig. 8) based on the Chinese standard^31^.

### Characterization

From each treatment group, four samples were sputter-coated with gold layer, and the morphology of wood sample surface was characterized by the scanning electron microscopy (SEM, FEI, and Quanta200). The Fourier transform infrared spectroscopy (FTIR, Thermo Fisher Scientific, and Nicolet 6700) measurements were used to explore the chemical changes. The wood samples were examined in the range of 4000–400 cm^−1^ with a resolution of 4 cm^−1^, scanning 32 times for each spectrum. The crystalline structure was analyzed by the X-ray diffraction (XRD, Philips, and D/max2200) operating with Cu radiation and at the acceleration voltage of 40 kV, the current of 30 mA, the scanning range (2θ) from 5 to 70°, and the scan rate of 4°/min.

### Data availablility statement

All data generated or analyzed during this study are included in this published article.
